# Chlorhexidine varnishes effectively inhibit *Porphyromonas gingivalis* and *Streptococcus mutans* — an *in vivo* study

**DOI:** 10.4103/0972-124X.75913

**Published:** 2010

**Authors:** Ashwin Mathew George, Suresh Kumar Kalangi, Mithuna Vasudevan, N. R. Krishnaswamy

**Affiliations:** *Department of Orthodontics, Ragas Dental College, Chennai, Tamil Nadu, India*

**Keywords:** Chlorhexidine varnish, *Porphyromonas gingivalis*, *Streptococcus mutans*

## Abstract

**Background:**

Chlorhexidine varnish (Cervitec- Ivoclar Vivadent- Liechtenstein) is a sustained-release delivery system that can provide protection against white spots and gingivitis, which are common iatrogenic side effects of orthodontic treatment. Chlorhexidine in varnish form does not depend on patient compliance, does not stain teeth or alter taste sensation like the mouth rinse.

**Materials and Methods:**

A split-mouth technique was followed in the treatment of 30 patients selected by stringent selection criteria, evaluating a single application of the test varnish on two randomly allotted quadrants along with a placebo on the other two quadrants. *Streptococcus mutans* counts responsible for white spots and *P. gingivalis* count [using PCR test] responsible for gingivitis were done at the start of the study, and then 1 and 3 months later.

**Results:**

The chlorhexidine varnish reduced the *Streptococci mutans* count at the end of 1 month, and this reduction was statistically significant. At the end of 3 months, there was no difference in the *S. mutans* counts between the two groups. There was a statistically significant reduction in the *P. gingivalis* count at the end of both 1 and 3 months in comparison to the placebo group.

**Conclusion:**

Chlorhexidine varnishes are capable of reducing *S. mutans* and *P. gingivalis* and *gingivitis*, thus improving the overall oral health of the patient. The side effects of chlorhexidine mouth rinses are not seen with this varnish. An application schedule of at least once a month is recommended as the effectiveness is reduced comparatively at the end of 3 months.

## INTRODUCTION

The effects of orthodontic treatment on the periodontium have been well documented.[1] Difficulty in institution of an adequate oral hygiene program and subsequent gingival inflammation is a commonly observed phenomenon following placement of orthodontic appliances. In addition, it has also been reported that placement of bands, brackets may lead to a change in the microflora, leading to an increase in the levels of periodontopathogenic organisms like *Porphyromonas gingivalis*.

Decalcification or white spot formation resulting from an imbalance between demineralization and remineralization of enamel has also been frequently reported to be associated with orthodontic treatment. *Streptococcus mutans* is found to be associated with the initiation and development of caries, and a significant increase in salivary and plaque levels is seen as early as the first week after placement of the appliance.[2]

This study was undertaken to evaluate the effect of Cervitec varnish [chlorhexidine and thymol] on *Streptococcus mutans* in supragingival plaque and *Porphyromonas gingivalis* in subgingival plaque.

A split-mouth technique was followed in the treatment of 30 patients selected by stringent selection criteria, evaluating a single application of the test varnish on two randomly allotted quadrants along with a placebo on the other two quadrants. *Streptococcus mutans* count responsible for white spots and *Porphyromonas gingivalis* count [using Polymerase chain reaction (PCR) test] responsible for gingivitis were done at the start of study, and then 1 and 3 months later.

Plaque sample collection (supragingival and subgingival) and evaluation of gingival condition was carried out at three different times:

* Baseline (T=0) — prior to varnish application
* One month (T=1) after varnish application
* Three months (T=3) after varnish application

Gingival parameters like gingival index by Ramfjord, plaque index by Silness and Loe and bleeding index by Muhlemann were also assessed at these times.

## RESULTS

### Evaluation of *S. mutans* count

**Figure 1:**
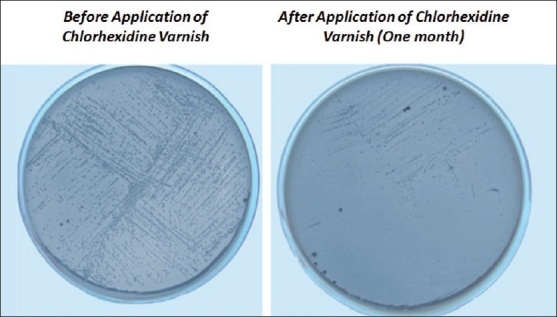
M. S. B. agar with colonies of *Streptococcus mutans* before application of chlorhexidine varnish and 1 month after application of chlorhexidine varnish

Chlorhexidine varnish reduced *Streptococcus mutans* count at the end of 1 month (T1), and this reduction was statistically significant as shown in Table 1 and Figure 1. At the end of 3 months, there was no difference in the *mutans* count between the two groups.

**Table 1:** *Streptococcus mutans* count in supragingival plaque

Time	Group	Number of Samples	Mean CFU (Log) /Ml	Std. Dev.	*P* value	Inference
T0	Placebo	30	3.7103	0.3882	0.208	Not significant
	Chlorhexidine	30	3.8446	0.4271		
T1	Placebo	30	3.8142	0.4258	0.026	Significant
	Chlorhexidine	30	3.5659	0.4187		
T3	Placebo	30	3.9448	0.4643	0.252	Not significant
	Chlorhexidine	30	3.8088	0.4465		

T0= Baseline, prior to varnish application; T1= One month after varnish application, T3= Three months after varnish application

### Evaluation of *Porphyromonas gingivalis* count

**Figure 2:**
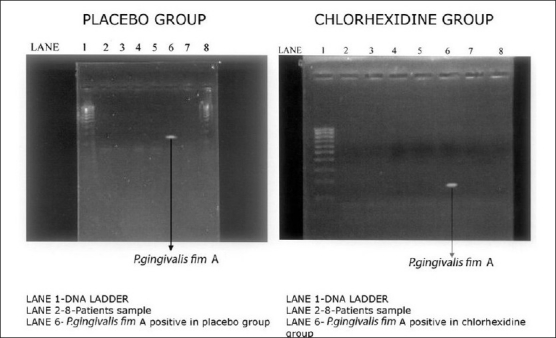
Expression of *P. gingivalis* fim A before varnish application

**Figure 3:**
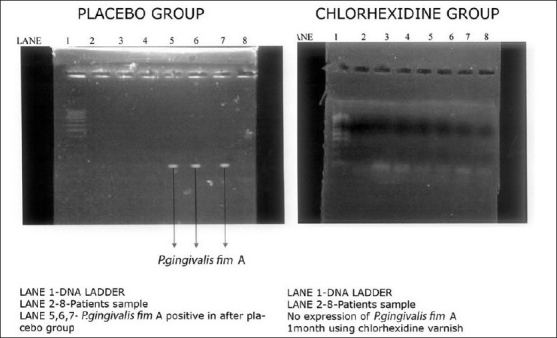
Expression of *P. gingivalis* fim A 1 month after varnish application

**Figure 4:**
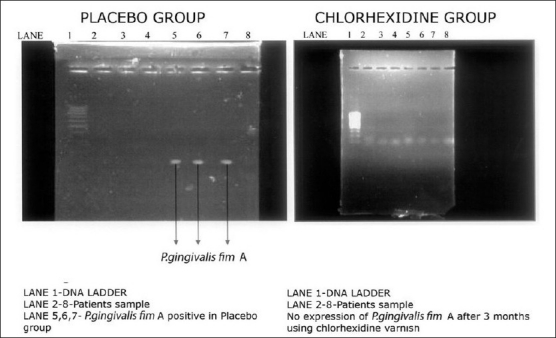
Expression of *P. gingivalis* fi m A 3 months after varnish application

There was a statistically significant reduction in the *Porphyromonas gingivalis* count at the end of one (T1) month and three (T3) months in the chlorhexidine varnish group in comparison to the placebo group as shown in the Table 2 and Figures 2–4

**Table 2:** *Porphyromonas gingivalis* (Fim A) expression in subgingival plaque

Time	Group	Numbers of Samples	*P. Gingivalis* (Fim A)	Percentage
T0	Placebo	30	1	6.66
	Chlorhexidine	30	1	6.66
T1	Placebo	30	3	20
	Chlorhexidine	30	0	0
T3	Placebo	30	3	20
	Chlorhexidine	30	0	0

### Evaluation of plaque index, gingival index and bleeding index

There was a statistically significant reduction in the scores on gingival index, plaque index and bleeding index in the chlorhexidine varnish group in comparison to the placebo group at the end of 1 month and 3 months as shown in Tables 3–5.

**Table 3:** Gingival index

Time	Gingival score	Groups		*P* value	Inference
		Placebo	Chlorhexidine		
T0	0	5	5	1.00	Not significant
	1	9	9		
	2	1	1		
T1	0	3	9	0.005	Significant
	1	7	5		
	2	5	1		
T3	0	0	10	0.00	Significant
	1	5	4		
	2	10	1		

**Table 4:** Plaque index

Time	Plaque score	Groups		*P* value	Inference
		Placebo	Chlorhexidine		
T0	0	0	4	1.00	Not significant
	1	10	10		
	2	1	1		
	3	0	0		
T1	0	1	9	0.001	Significant
	1	6	6		
	2	8	0		
	3	0	0		
T3	0	0	9	0.00	Significant
	1	2	6		
	2	11	0		
	3	2	0		

**Table 5:** Bleeding index

Time	score	Groups		*P* value	Inference
		Placebo	Chlorhexidine		
T0	0	6	7	1.00	Not significant
	1	9	8		
	2	0	0		
T1	0	5	12	0.025	Significant
	1	10	3		
	2	0	0		
T3	0	3	13	0.001	Significant
	1	12	2		
	2	0	0		

## DISCUSSION

Over the past few decades, chlorhexidine has evolved into the gold standard among the antimicrobial substances used in dentistry. Chlorhexidine and thymol (Cervitec varnish) interact and adhere to the pellicle proteins or other constituents, establishing a reservoir from which chlorhexidine can be slowly released over time. Both molecules also react with the salivary proteins and salivary bacteria, thus participating in the reduction of the amount of plaque, and of the count of *S. mutans*.

The results of this study indicated that a single application of the test varnish was capable of significantly reducing *S. mutans* count and *Porphyromonas gingivalis* count at the end of first month. However, it was noted that the test varnish had no effect on *S. mutans* at the end of 3 months.

*Porphyromonas gingivalis* levels were, however, significantly lower even at the end of 3 months. Recent studies have evaluated the effect of supragingival plaque on subgingival biofilm formation and shown a direct and positive correlation.[3] As the plaque scores were significantly lower in the test group, the *P. gingivalis* levels were correspondingly low. The importance of co-aggration in biofilm formation has been previously documented, with *P. gingivalis* being shown to co-aggregate with several oral streptococci, especially S. gordonii of supragingival plaque.[4] A significant reduction in the levels of these organisms as a result of decreased supragingival plaque could have contributed to the observed reduction in *P. gingivalis* count.

*Porphyromonas gingivalis* has been recently described as a host-associated pathogen, and its role in initiation of periodontal disease is well recognized.[5] It has also been shown that chlorhexidine reduces the level of inflammatory mediators; and also reduces the amount of gingival crevicular fluid, which is an indicator of inflammation. This effect of Cervitec on the gingival crevicular fluid and archidonic acid metabolites PGE2, PGI2 and LTB4 could also be the reason for the improvement of gingival health as seen till the end of 3 months.

## CONCLUSION

Chlorhexidine varnishes are capable of reducing *Streptococci mutans, Porphyromonas gingivalis* and gingivitis, thus improving the overall oral health of the patient.

The side effects that are seen with the long-term use of chlorhexidine mouth rinses are not seen with this new mode of delivery, viz., chlorhexidine application in varnish form. Chlorhexidine application in varnish form does not depend on patient compliance; does not stain teeth, tongue, the mucosa and composite restoration or alter taste sensation.

An application schedule of at least once a month is recommended, as the effectiveness is reduced comparatively at the end of 3 months, especially when controlling *S. mutans* responsible for white spot lesions.
